# The effect of Active Plus, a computer-tailored physical activity intervention, on cognitive functioning of elderly people with chronic illness(es) – study protocol for a randomized controlled trial

**DOI:** 10.1186/s12889-019-7517-3

**Published:** 2019-08-30

**Authors:** Esmee Volders, Catherine A. W. Bolman, Renate H. M. de Groot, Lilian Lechner

**Affiliations:** 1 Faculty of Psychology and Educational Sciences, Open University of the Netherlands, 6419 AT Heerlen, The Netherlands; 2 Welten Institute – Research Centre for Learning, Teaching and Technology, Open University of the Netherlands, 6419 AT Heerlen, The Netherlands; 3 Nutrition and Translational Research in Metabolism (School NUTRIM), Maastricht University, 6200 MD Maastricht, The Netherlands

**Keywords:** Physical activity, Cognition, Executive functions, Older adults, Chronic illness, Physical activity promotion

## Abstract

**Background:**

Physical activity not only is beneficial to a person’s health, but can also have a positive influence on cognitive functioning. However, elderly people with chronic illness(es) often do not meet the physical activity guidelines. Physical activity programs for the elderly exist, but these are often expensive and not easily accessible to the elderly with chronic illness(es). In addition, the beneficial effects of these physical activity programs on cognitive functioning have never been specifically tested in this target group. Hence, this randomized controlled trial aims to test whether Active Plus, a proven effective physical activity intervention, is able to improve the cognitive functioning of elderly people with chronic illness(es) or to slow down cognitive decline. In addition, it studies what kind of activity, intensity, duration and frequency of physical activity most strongly influence cognitive functioning.

**Methods:**

A randomized controlled trial is performed, comparing the Active Plus intervention group to a waiting list control group. In total 540 older adults (≥65 years) with at least one chronic illness that limits mobility are recruited from 7 municipalities. Comparable neighborhoods within a municipality are randomly allocated to the intervention or control group. Baseline and follow-up measurements after 6 and 12 months assess cognitive functioning and physical activity behavior, measured both objectively with an accelerometer and subjectively with a self-report questionnaire. Multilevel analyses are conducted to assess effects on cognitive functioning, including analyses on moderation effects for physical activity type, frequency, duration and intensity.

**Discussion:**

To our knowledge this is the first study to investigate effectiveness of a physical activity program on cognitive functioning in elderly people suffering from a broad range of chronic illnesses. If proven effective Active Plus would be a very cost effective intervention not only to increase physical activity, but also to improve cognitive functioning or slow down cognitive decline. Up till now clear evidence is lacking on the kind of physical activity, intensity, duration and frequency needed to achieve cognitive benefits. By measuring both with accelerometers and self-report questionnaires we hope to gain insight in these processes.

**Trial registration:**

Nederlands Trial Register NL6005; Date of Registration 21-03-2017.

## Background

Chronic illnesses in elderly people have a major impact on society. The older a person becomes, the more likely they develop one or more chronic illnesses, such as cardiovascular disease, osteoarthritis and diabetes [1–4]. These chronic illnesses are associated with, among others, depressive symptoms, cognitive functioning problems, and mobility limitations [5–7]. Eighty percent of the elderly population living in developed countries suffer from a chronic illness and 50–75% have to deal with multiple chronic illnesses [4, 8, 9]. Therefore, the demand for healthcare among elderly people is large and will increase even further in the future due to the aging world population [3]. Most countries experience growth in the number and proportion of older people in their population. The number of people aged 65 years or over is expected to nearly triple by 2050, rising from 542 million globally in 2010 to about 1.5 billion in 2050 [10]. More importantly, worldwide the population aged 60 or over is growing faster than all younger age groups. Since the number of elderly people increases and the potential labor force remains the same or even declines, the costs of health care will increase [11].

Chronic illnesses in the elderly not only have a major impact on society, but also on their individual degree of independence and quality of life [12]. Elderly people may suffer not so much from the illnesses themselves, but from the limitations as a result of these chronic illnesses [13]. These limitations, such as mobility restrictions, stand in the way of an independent life and can lead to loneliness [14]. Consequently, the World Health Organization [15] focuses its policy framework on active aging by which chronic illnesses and reduced mobility should be prevented on the one hand, and vitality, self-reliance, participation in society and quality of life should be optimized on the other hand [3]. This should culminate in elderly people living independently for as long as possible; and good health is a prerequisite for this [16]. The functioning of the elderly can be optimized by a change in lifestyle, of which physical activity (PA) is an important component [3].

Engaging in regular PA has great health benefits [17]. It is effective in controlling weight, strengthening muscles and bones, improving mood and the ability to do daily activities and preventing falls. Additionally, there is indisputable evidence of the effectiveness of regular PA in the prevention of several chronic diseases (e.g., cardiovascular disease, diabetes, cancer, hypertension, obesity, depression and osteoporosis) and premature death [18].

Furthermore, there is preliminary evidence to suggest that PA is beneficial for cognitive functioning (CF). An increasing number of scientific studies show that PA has a positive influence on CF in general [19]. However, not all PA programs show this result [20, 21]. In the aging brain, sufficient PA correlates with a reduced risk for cognitive decline [22]. Several review articles state that executive functions appear to benefit most from PA [23–25]. Executive functions are higher-order cognitive processes that are necessary to control cognitive behavior. These processes include planning, working memory, inhibition, mental flexibility, as well as the initiation and monitoring of action [26]. Without these functions, well-organized behavior is not possible. However, the underlying mechanisms on how PA may protect against cognitive decline are still somewhat unclear, although elevated neurotrophin levels, improved vascularization, facilitation of synaptogenesis, decreased systemic inflammation, and reduced disordered protein deposition may play a role [19]. Studies have shown that adding any moderate intensity PA program in later adulthood is beneficial for cognitive performance, especially for very sedentary older adults [27]. All things considered, by engaging in regular PA, one can therefore actively contribute to a healthy brain.

Notwithstanding the increasing body of evidence for the importance of regular PA, most elderly people do not reach the recommended guidelines [28]. These guidelines state that adults should be active for at least 150 min at a moderate-intensity spread over a few days per week and perform resistance training at least 2 days per week. Data from the U.S. Department of Health and Human Services indicate that more than 80% of adults do not meet these guidelines for both aerobic and muscle-strengthening activities [29]. Comparable numbers are shown in a study in the Dutch population; only 33% of the elderly of 65 years or over meet the guidelines [30].

Within the elderly population those with chronic illnesses are most sedentary and perceive many PA related barriers [31]. Although a few PA programs exist for elderly with chronic illnesses (e.g. Coach2Move [32], Strong-for-Life [33], Life-P [34]) most programs are not easily accessible, often not attended by elderly with chronic illnesses and often only reaching already active elderly [35]. Moreover, the programs are often face-to-face and high demanding [36].

Active Plus is an existing proven effective personalized PA program for elderly people which provides the target group with 3 personalized PA advices (online and print delivered) in 4 months [37]. Preceded by a questionnaire (e.g. on current PA and perceived PA beliefs and barriers) a computer-tailoring program generates personalized advice, tips and exercises that are sent to the user. Active Plus raises awareness of PA, and guides PA initiation and maintenance. The program is tailored to patient and disease specific situations and needs [38]. Previous research in a general population of people aged over 50 years showed in the Active Plus group in 1.5 h per week more moderate to vigorous intensity PA after 1 year compared to controls [37, 39, 40], even in elderly people with limited mobility [41, 42]. Active Plus is highly cost-effective, reduces disease incidence and potentially reaches many elderly people with a chronic illness and limited mobility at very low costs [43–45].

As the program Active Plus has strong indications for long-term effects on moderate to vigorous PA in the elderly with chronic illnesses and limited mobility, we hypothesize that Active Plus improves cognitive functioning in this target group. In a meta-analysis and systematic review on effects of exercise on cognitive function in chronic disease patients by Cai [46] a positive overall effect of exercise interventions on cognitive function was found. However, 22 out of 35 included studies only included patients with Alzheimer’s disease or Mild Cognitive Impairment. Other included studies targeted their intervention to only one individual chronic illness (i.e. cancer, heart failure). To our knowledge, the effects of PA on CF in an elderly population which suffers from a broad range of chronic illness(es) have not yet been tested. Furthermore, the interventions included in the meta-analysis were site-situated, which is high demanding, more expensive, and mainly focused on exercise, while a computer-tailored PA intervention like Active Plus has a strong focus on stimulating daily PA [35, 36]. Therefore, the primary objective of the present Active Plus study is to investigate the short-term (6 months) and long-term (12 months) effects of the Active Plus program on CF of people aged 65 years or older with chronic illness(es) through a clustered randomized controlled intervention trial (RCT). This paper describes the protocol for this objective and the other objectives below.

PA is complex behavior consisting of type of activity, duration, frequency, and intensity [47]. There are some indications that the beneficial effects of PA on CF are independent of these characteristics of PA [46]. However, another meta-analysis [48] showed that only a duration of > 45 min to ≤60 min per session at a moderate-to-vigorous intensity on as many days per week was beneficial for CF. Therefore, it is unclear what characteristics PA should have to be effective on CF in clinical practice. To provide more insight in this matter, we will study as another primary objective the relationship between the necessary type, frequency, duration and intensity of PA to increase CF or slow down its decline in the elderly with chronic illness(es) (ECI). To test PA behavior, we use both self-report questionnaires and accelerometers. Self-report questionnaires are known for overestimating PA, however they measure different constructs than accelerometers [49]. Therefore, both ways of measuring PA are administered in this project.

As mentioned before, elderly people with chronic illnesses have a lesser individual degree of independence and quality of life [12], and experience more feelings of loneliness [14]. Sufficient levels of PA and CF are important for self-reliance and a good health related quality of life (HRQoL) [3]. However, being sufficiently physically active is a great challenge for this target group [31]. Therefore, a secondary objective is to study the effects of the Active Plus intervention on self-reliance, HRQoL and loneliness.

## Methods

### Study design

The study is a parallel, two-group cluster-RCT with a waiting list control group and assessments at baseline, six and 12 months. Due to the nature of the study, it is not possible to blind the researchers. Participants do not know there are two groups being examined. Ethical approval for the study was obtained from the Research Ethics Committee (cETO) of the Open University and the trial is registered with the Dutch Trial Register, protocol number NL6005. This protocol is reported in accordance with SPIRIT guidelines. The study is funded by a grant from the Brain Foundation Netherlands. All participants provide written informed consent prior to commencing the study. Participants may withdraw from participation at any time without giving an explanation, no data will be collected anymore. There are no interventions or care prohibited during the trial.

### Participants

#### Recruitment

Five hundred and forty (540) participants are recruited over a period of 6 months through an invitation letter by one of the seven cooperating municipalities. Each municipality will select two groups for participation, who originate from one or more comparable residential areas or neighborhoods based on their socioeconomic status. Neighborhood selection is based on data of www.waarstaatjegemeente.nl, a website where municipalities can compare their own data with other municipalities on themes such as youth aid, finances and sustainability. These comparable neighborhoods are randomly assigned to either the experimental group or the waiting list control group [50]. Names and addresses of independently living men and women aged 65 years or older residing in these municipalities are drawn from the records of the residents’ registration office taking the General Data Protection Regulation of the European Union into account. The number of persons drawn from the records varied between 500 and 4000 per municipality.

#### Eligibility criteria

Participants must be 65 years or older, be fluent in Dutch and suffer from at least one chronic illness that affects mobility (e.g. musculoskeletal and back disorder, COPD, rheumatism, osteoporosis, chronic heart disease) or other physical conditions (e.g. visual or hearing impaired) that may affect mobility. Participants with self-reported cognitive problems or wheelchair users are excluded from the study. Participants must be able to walk at least 100 m, possibly with the help of a rollator or walking stick.

#### Procedure

**Fig. 1:**
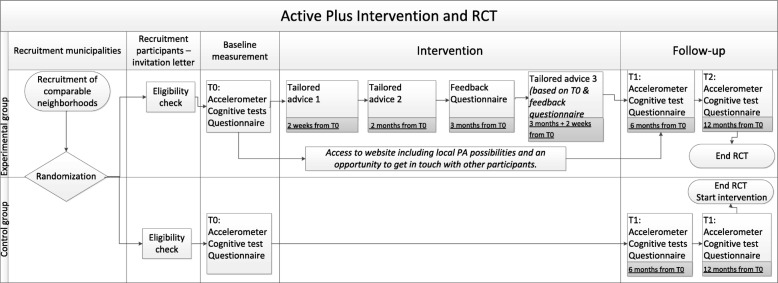
Schematic overview of the randomized controlled trial procedure and the intervention

Figure 1 provides an overview of the study procedure. Residents from both the selected experimental and the waiting list control neighborhoods receive an invitation letter and an informed consent from their municipality. The informed consent contains a list with the most common chronic conditions that affect mobility with which interested residents can check their eligibility. When interested residents suffer from at least one chronic condition, they can send their informed consent to the researchers. Subsequently, eligibility criteria are checked in the informed consent and by a phone call with the research assistant. With all eligible persons an appointment is set for the cognitive functioning test.

Before the CF test, participants in both groups receive an accelerometer device by mail with an explanation on how and when to wear it. They are asked to wear it for 7 consecutive days on their right hip. The day after the last wearing day they are visited at home by a researcher (assistant) or student who brings the accelerometer back to the research institute.

The CF tests are assessed by a trained researcher or student on an iPad Air 2 at the participants’ home with Inquisit 5 software [51]. To become familiar with the iPad, participants are asked to draw a house and a tree in the Notes application. The CF tests start with the first part of the 15 Words Learning Task, a Dutch adaptation of the Rey’s Verbal Learning Task, followed by the Trail Making Test part A and B, the Stop Signal Task, and the Letter Digit Substitution Task. The test ends with the second part of the 15 Words Learning Task. The total test takes around 45 min to complete. Any occurring disturbances or difficulties in completing the test are administered. For more information on the CF tests see the outcome measures section.

After the CF test participants choose to complete the baseline questionnaire online on www.actief-plus.nl or on paper (with a prepaid return envelope). Thereafter, the 4 month lasting intervention commences for the experimental group. After 6 months and 12 months participants are contacted again to make an appointment for the second and third measurement. They wear the accelerometer device again, are visited at home to take the cognitive tests and complete a questionnaire. Procedures for the second and third measurement are similar to the first measurement. After the final assessment participants in the control group receive access to the Active Plus intervention.

#### Experimental condition

The computer tailored Active Plus intervention stimulates PA among people aged 65 or over with a chronic illness and is proven to be effective in increasing PA behavior [37, 42–45]. The intervention is systematically developed using the Intervention Mapping Protocol, and based on various theoretical models such as the Theory of Planned Behavior [52], Precaution Adoption Process Model [53], the Integrated Model for Change (I-Change Model) [54], and a self-regulation model [55]. The intervention intents to take up and sustain PA behavior by influencing (pre−/post-) motivational constructs. The pre-motivational constructs addressed are awareness and knowledge among others. Constructs such as intention, social influence, intrinsic motivation, attitude and self-efficacy are addressed in the motivational stage. Post-motivational constructs include action planning, coping planning, strategic planning, commitment, and self-regulation skills. Especially for the targeted population of ECI tailored information is added on several chronic illnesses, the risks of sedentary behavior, and the benefits of PA for CF.

Participants in the experimental group receive advice on three occasions (see Fig. 1), tailored to the answers they give in questionnaires. The first personal advice is delivered right after the baseline assessment. Participants receive the second advice 2 months after the baseline questionnaire. And within 4 months after the baseline questionnaire feedback on progress in PA behavior is given, based on the second assessment (only used to generate a third advice). All participants in the experimental condition receive a copy of their personal advises by email (if they have registered an email address) and by mail as well. Participants receive an invitation for the follow-up assessment by mail, together with the follow-up questionnaire and a prepaid return envelope, and by email, including a link to the questionnaire.

The intervention gives tailored information on PA and its determinants especially for older adults with chronic illness(es), such as the importance of PA to maintain adequate cognitive functioning. The exact content of the advices depends on the participants’ personal characteristics (e.g. age, gender, and presence of a chronic condition) and psychosocial characteristics (e.g. the (pre−/post-) motivational constructs as described above), their current PA behavior, and to what extent they are willing to alter their behavior. Each participant in the experimental group receives all the tailored information, although the participants current PA behavior and stage of change controls in which advice the information is given. The intervention is integrated into a website where additional information on local PA possibilities (e.g. walking or cycling routes in their own neighborhood or sport clubs) can be found, as well as a user forum, and examples of PA exercises.

#### Waiting list control condition

Participants allocated to the waiting list control condition do not have access to the personalized PA advises, the additional information, user forum or examples of PA exercises until the end of the 12 month study period. As such, they have no access to any of the intervention features, and are encouraged to continue with their usual activities. After the study period they receive their personalized PA advises.

### Outcomes

As it is the main goal of the current project to test if the Active Plus program is able to increase CF or to slow down its decline by means of sufficient PA, the primary outcomes are CF and PA. Secondary outcome measures are self-reliance, health related quality of life, and loneliness. Outcomes are assessed at baseline, and after six and 12 months.

#### CF outcomes

The Verbal Learning Test (VLT) [56] is a frequently used measure of verbal memory. Several studies found PA interventions effective in improving memory [19]. As memory complaints are often the first cognitive complaints people suffer from this aspect of cognitive functioning is also included in the current study. In this test, 15 words are presented one by one on an iPad screen in fixed order, with a presentation time of 1 s and an interstimulus interval of 1 s. Afterwards participants are asked to verbally recall the words they have remembered. The first trial is followed by four more trials in which the words are presented in identical order. After a delay of 15–25 min in which the remaining CF tests are assessed, and unexpectedly for the participants, the instruction is given to recall the 15 words learned once more. Finally, a recognition trial is administered. Greater test scores indicate better verbal memory.

The Letter-Digit Substitution Test (LDST) [57] is a modification of the procedurally identical Symbol-Digits Modalities Test [58]. This test is often used as a measure of processing speed of general information, which is found to be affected by PA [20]. In the LDST, letters are presented and the participant has to respond by clicking the corresponding digits, according to a key. After a practice round of 10 letters, the participant has 60 s to replace the randomized letters with the appropriate digit indicated by the key. Greater test scores indicate better speed of information processing.

The Trail Making Test (TMT) provides information on visual search, scanning, speed of processing, mental flexibility, and executive functions [59]. The TMT consists of two parts. TMT-A requires the participant to draw lines with their finger sequentially connecting 25 encircled numbers distributed on the iPad. Task requirements are similar for TMT-B except the participant must alternate between numbers and letters (e.g., 1, A, 2, B, 3, C, etc.). The score on each part represents the amount of time required to complete the task. Score on B-A is a measure for shifting, which is a construct of executive functioning and is found to be affected by PA [60]. The larger the score B-A is, the more difficulty the participant has with shifting.

The Stop Signal Test is a useful test for the investigation of response inhibition which is a construct of executive functioning and is affected by PA [60, 61]. The task is to press the left response key if the arrow on the iPad screen points to the left and press the right response key if the arrow points to the right unless a signal beep is played after the presentation of the arrow. In this case the response should be stopped before execution (inhibition). The test consists of two parts: The first part is a practice block of 32 trials. Afterwards, three blocks of 64 trials are taken with 10 s of rest in between blocks. The delay between presentation of arrow and signal beep, starting at 250 milliseconds (ms), is adjusted up or down by 50 ms depending on performance. The delay gets longer if the previous signal stop was successful (up to 1150 ms) and it gets smaller if the previous signal stop was not successful (down to 50 ms). Thus, the task uses a staircase design for the stop signal delay (SSD), allowing the task to adapt to the performance of the participant, narrowing in on the 50% success rate for inhibition. Outcome measure is an estimation of the stop signal reaction time in ms (SSRT), which is the time required to stop the initiated go-process. The slower the SSRT the more difficult to stop the go-process.

#### PA outcomes

Self-reported PA is measured using the validated Short Questionnaire to Assess Health Enhancing Physical Activity (SQUASH) [62], assessing activities regarding household and leisure time and sports. For each activity the frequency, the duration and the intensity is administered. The SQUASH questionnaire classifies PA into light (metabolic equivalent (MET) < 3.0), moderate (MET 3.0–5.9), and vigorous (MET > 6) [63]. The scoring manual is used to calculate these constructs and to exclude any extreme values. The SQUASH has reasonable reliability (ρ = .58) and validity (ρ = .45) opposed to an accelerometer [62].

PA is also measured using the ActiGraph GT3X-BT (ActiGraph, Pensacola, FL). Participants wear the accelerometer on an elastic belt on their right hip for 7 days. During the night participants are not obliged to wear the device. Participants are asked to remove the devices while showering or swimming. Data is downloaded and analysed using ActiLife software. Measurements are considered valid if there is at least 4 days of data with at least 10 h of wear time per day [64]. Non-wear periods are eliminated from the analyses and are identified with the Choi algorithm [65]. The Choi algorithm identifies 90 min of consecutive zero counts as non-wear time, which may be interrupted by a maximum of 2 min of non-zero counts. To distinguish between light, moderate and vigorous PA the software uses data from 3 axes based on 60 s epochs. Freedson-VM cut-off points, developed by Sasaki [66], and the cut-off points developed by Aguilar-Fariaz [67] are used as algorithms to determine the average hourly and daily time spent per metabolic rate.

#### Secondary outcomes

Self-reliance is measured with the WHODAS II [68]. This questionnaire is based on an international classification system and is used when assessing the level of functioning of a person with an emphasis on activities and participation level. The WHODAS II measures the following domains: understanding and communicating, mobility, self-care, interaction with people, household and activities, and participation in society. The questionnaire focuses on the last 30 days and consists of twelve items. Higher scores indicate a lower self-reliance. Test-retest studies of the 36-item scale in countries across the world found it to be highly reliable [68].

Health Related Quality of Life is measured with the SF-12v2 questionnaire which measures eight health domains to assess mental and physical health and consists of twelve questions [69]. Mental health-related scales include vitality, social functioning, role emotional, and mental health. Physical health-related domains include general health, physical functioning, role physical, and body pain. A high score corresponds to a better health condition. The instrument has been validated across a number of chronic diseases and conditions [69, 70].

Experienced feelings of loneliness are measured with de Jong Gierveld Loneliness Scale [71]. The questionnaire is a reliable and valid measurement instrument for overall, emotional, and social loneliness and consists of six items using a 10-point scale, ranging from 1 (not lonely) to 10 (extremely lonely).

#### Other relevant measures

Demographic and chronic disease related characteristics including age, gender, marital status, body mass index (BMI), educational level, income, degree of limitation regarding chronic illness(es) are assessed. BMI is defined as body mass divided by the square of body height. Educational level is categorized into low (i.e. primary, basic vocational, or lower general school), moderate (i.e. medium vocational school, higher general secondary education, and preparatory academic education), or high (i.e. higher vocational school or university level) according to the Dutch educational system. The degree of impairment is measured with a self-report questionnaire [38]. The participant states for 14 common chronic illnesses (i.e. cardiovascular, osteoarthritis) and physical conditions (i.e. hearing or visually impaired) to what degree he/she is limited in his/her PA behavior by one of the mentioned diseases or by another not mentioned disease. For each chronic illness, the participant scores the degree of impairment on a 5-point scale ranging from 0 = not applicable, 1 = not at all/hardly, 2 = a little, 3 = very, to 4 = extremely.

#### Sample size and statistical power

Sample size calculations are based on the primary outcome measures of CF. Based on literature on the effects of PA on CF (i.e. Memory, Set shifting, Inhibition of pre-potent responses and Processing Speed) an effect size (ES) of .30 is expected on the cognitive outcomes. Northey [48] conducted a meta-analysis on the effects of exercise interventions on cognitive function in adults older than 50. They found an overall ES of .29; when only participants are included without Mild Cognitive Impairment, the ES rose up to .36. Colcombe & Kramer [23] reported ES of almost .5 of fitness effects on cognitive functions in older adults. A meta-analysis of Chang [72] reported a lower ES = .10, though for all age groups, suggesting that the effect on cognitive performance in elderly is larger (ES = .18) than in young people. Therefore, we estimated an ES of 0.3 for our primary outcome measures of CF. An moderate ES (≥.30, Cohen [73]) is considered clinically relevant for practice.

The needed sample size has to be inflated to take account of the multilevel design. Therefore, an estimate of Intra Cluster Correlation (ICC) is used, based on the ICC of the previous Active Plus projects (ICC < .01). Statistical power analysis using G*Power [72] (ES = .30; power = .80; ICC = .01) showed that 190 participants per group are required. Based on our previous studies [45] we expect a 30% dropout rate at 12 months. We therefore will include 270 participants per group.

#### Statistical analyses

Independent t, Mann-Whitney U, and Chi-square tests are used to determine differences in baseline characteristics between the experimental group and the waiting list control group. To assess predictors of dropout, logistic regression with condition, baseline outcome measures, demographics, and degree of limitation regarding chronic conditions will be performed. Depending on the distribution of continuous, categorical, and interval outcomes, appropriate distribution and relevant statistical models will be used. These multilevel models will assess intervention effects on CF at end-of-intervention (6-months and 12-months) as well as the difference between 6- and 12-months measurements. To identify significant covariates, bivariate Pearson correlations are conducted between demographic variables and the independent and dependent variables of interest. Moderation analysis of type, duration, frequency and intensity of PA will be conducted. The most appropriate procedure for handling missing data will be selected after inspecting the amount and pattern of missing data.

## Discussion

To our knowledge, this is the first study that will investigate the effectiveness of an online computer tailored PA program on CF in people aged 65 years or older who suffer from a broad range of chronic illness(es). Until now, most research on the effects of PA on CF is done with people who suffer from one specific chronic illness and results are equivocal [46]. However, the population of people aged over 65 often suffer from a broad range of chronic illnesses and often have more than one illness [9]. Therefore, the results of this study are expected to be more generalizable to the general elderly population than previous research.

 The Active Plus intervention stimulates PA in daily life and takes place in a real life setting. We already know Active Plus is effective in preventing the development of somatic diseases (e.g. diabetes) [43]. The effects on CF of such an easily accessible program that focusses on PA in daily life are not tested yet, as opposed to strenuous exercise programs that are site-based. However, such programs would be very beneficial to broadly reach elderly with chronic illness(es). Until now, the interventions used to investigate the effect of PA on CF in elderly with chronic illness(es) are often intensive site-situated [35, 36]. It is unmanageable and financially unaffordable to approach the complete target group with intensive face-to-face programs [35, 36]. Active Plus would potentially be a very cost effective solution [43–45].

This study will not assess effects longer than 1 year after the baseline measurement and start of the intervention due to practical implications. However, it might be that cognitive effects of Active Plus are only visible after a longer timeframe, because it improves the cognitive reserve capacity and in this way prevents cognitive decline over a longer time. Nonetheless, other studies showed intervention effects on CF already after 6–12 months [19].

A great strength of this study is that the change in PA is measured both objectively with an accelerometer and with a self-report questionnaire. Questionnaires provide detailed insight regarding type of PA (e.g. household PA, leisure time PA), frequency and duration of specific PA behaviors. Such specific information is useful in targeting PA interventions and cannot be obtained from accelerometers. However, self-report PA questionnaires are known for their overestimation of PA which might occur due to misclassification of activities, double reporting, recall bias, and social desirability [74]. Accelerometers measure the quantity and intensity of movement. Although accelerometers result in objective PA measurements, they also have their limitations; they do not provide information on the type of activity and they are limited in the measurement of swimming/water-based activities, cycling, step/inclined activity, or strength exercises [74–77]. By using both methods we will gain optimal insight in type, frequency, duration and intensity of PA that is needed to increase CF or slow down decline of CF.

## Data Availability

Data sharing is not applicable to this article as no datasets were generated or analysed during the current study. Once available, study data are available from the corresponding author on reasonable request.

## Abbreviations

BMI: Body mass index
cETO: Research Ethics Committee of the Open University
CF: Cognitive functioning
ECI: Elderly people with chronic illness(es)
ES: Effect size
HRQoL: Health related quality of life
ICC: Intra cluster correlation
LDST: Letter digit substitution test
MET: Metabolic equivalent
Ms: Millisecond
PA: Physical activity
RCT: Randomized controlled trial
SQUASH: Short questionnaire to assess health enhancing physical activity
SSRT: Stop signal reaction time
SST: Stop signal test
TMT: Trailmaking test
VLT: Verbal learning test
